# Interactions between human immunodeficiency virus (HIV)-1 Vpr expression and innate immunity influence neurovirulence

**DOI:** 10.1186/1742-4690-8-44

**Published:** 2011-06-06

**Authors:** Hong Na, Shaona Acharjee, Gareth Jones, Pornpun Vivithanaporn, Farshid Noorbakhsh, Nicola McFarlane, Ferdinand Maingat, Klaus Ballanyi, Carlos A Pardo, Éric A Cohen, Christopher Power

**Affiliations:** 1 Department of Medicine University of Alberta, Edmonton, AB, T6G 2S2, Canada; 2 Department of Medical Microbiology & Immunology, University of Alberta, Edmonton, AB, T6G 2S2, Canada; 3 Department of Physiology, University of Alberta, Edmonton, AB, T6G 2S2, Canada; 4 Department of Clinical Neurosciences, University of Calgary, Calgary, AB, T2N 1N4, Canada; 5 Department of Pharmacology, Faculty of Science, Mahidol University, Rama IV road, Bangkok, 10400, Thailand; 6 Department of Neurology and Pathology, Johns Hopkins University School of Medicine, Baltimore, MD 21287, USA; 7 Institut de recherches cliniques de Montréal (IRCM) and Department of Microbiology and Immunology, Université de Montréal, 110, Pine Avenue, Montreal, Quebec, H2W 1R7, Canada

## Abstract

**Background:**

Viral diversity and abundance are defining properties of human immunodeficiency virus (HIV)-1's biology and pathogenicity. Despite the increasing availability of antiretroviral therapy, HIV-associated dementia (HAD) continues to be a devastating consequence of HIV-1 infection of the brain although the underlying disease mechanisms remain uncertain. Herein, molecular diversity within the HIV-1 non-structural gene, Vpr, was examined in RNA sequences derived from brain and blood of HIV/AIDS patients with or without HIV-associated dementia (HAD) together with the ensuing pathobiological effects.

**Results:**

Cloned brain- and blood-derived full length *vpr *alleles revealed that amino acid residue 77 within the brain-derived alleles distinguished HAD (77Q) from non-demented (ND) HIV/AIDS patients (77R) (*p *< 0.05) although *vpr *transcripts were more frequently detected in HAD brains (*p *< 0.05). Full length HIV-1 clones encoding the 77R-ND residue induced higher *IFN-α*, *MX1 *and *BST-2 *transcript levels in human glia relative to the 77Q-HAD encoding virus (*p *< 0.05) but both viruses exhibited similar levels of gene expression and replication. Myeloid cells transfected with 77Q-(p*Vpr77Q-HAD*), 77R (p*Vpr77R-ND*) or Vpr null (p*Vpr*^*(-)*^)-containing vectors showed that the p*Vpr77R-ND *vector induced higher levels of immune gene expression (*p *< 0.05) and increased neurotoxicity (*p *< 0.05). Vpr peptides (amino acids 70-96) containing the 77Q-HAD or 77R-ND motifs induced similar levels of cytosolic calcium activation when exposed to human neurons. Human glia exposed to the 77R-ND peptide activated higher transcript levels of *IFN-α*, *MX1*, *PRKRA *and *BST-2 *relative to 77Q-HAD peptide (*p *< 0.05). The Vpr 77R-ND peptide was also more neurotoxic in a concentration-dependent manner when exposed to human neurons (*p *< 0.05). Stereotaxic implantation of full length Vpr, 77Q-HAD or 77R-ND peptides into the basal ganglia of mice revealed that full length Vpr and the 77R-ND peptide caused greater neurobehavioral deficits and neuronal injury compared with 77Q-HAD peptide-implanted animals (*p *< 0.05).

**Conclusions:**

These observations underscored the potent neuropathogenic properties of Vpr but also indicated viral diversity modulates innate neuroimmunity and neurodegeneration.

## Background

Human immunodeficiency virus type 1 (HIV-1) infection is a global health problem for which the pathogenic mechanisms causing disease occurrence and the acquired immunodeficiency syndrome (AIDS) are incompletely understood [1-5]. HIV infection of the brain is a major component of HIV-associated disease burden because of the brain's comparatively privileged sites for viral replication and persistence; moreover, the brain is relatively inaccessible to many antiretroviral therapies [6-8]. HIV-associated dementia (HAD) is caused by infection of the brain with ensuing glial activation and neuronal damage and death, characterized by motor, behavioral, and progressive cognitive dysfunction [9]. The prevalence of HAD is approximately 5-10% in antiretroviral therapy-exposed populations. HAD arises due to both pathogenic host responses, mediated by infected and activated microglia and astrocytes, as well as the cytotoxic properties of viral proteins in susceptible individuals [10-14]. Among the expressed viral proteins, viral protein R (Vpr) has garnered increasing attention because of its importance in terms of modulating HIV infection of macrophages, regulation of cell cycle pathways and its pro-apoptotic actions [15-19]. Vpr causes neuronal apoptosis through disruption of mitochondrial function [20-22].

Molecular diversity is one of HIV's defining properties, which has precluded the development of effective anti-HIV vaccines but also contributes to the emergence of both virulent and drug-resistant viral strains [23-25]. Among blood-derived HIV sequences, Vpr exhibits molecular diversity although the mechanistic consequences of these sequence differences are unclear but appear to be associated with clinical phenotypes in some circumstances [26-29]. Given these circumstances including Vpr expression and potential pathogenic actions in the brain together with its capacity to mutate in conjunction with clinical phenotypes, it was hypothesized that Vpr might show molecular diversity in the brain, influencing its functions as a neurotoxic ligand or a pathogenic modulator of neuroinflammation [30-32]. Herein, brain-derived HIV-1 Vpr sequences exhibited a consistent mutation, which distinguished non-demented (ND) from demented (HAD) HIV/AIDS patients; the molecular motif within Vpr associated with dementia was less neuropathogenic but also exerted blunted anti-viral and neurotoxic host responses, providing a new perspective into HIV-associated neurovirulence.

## Results

### HIV-1 vpr sequence diversity in brain and blood

**Figure 1:**
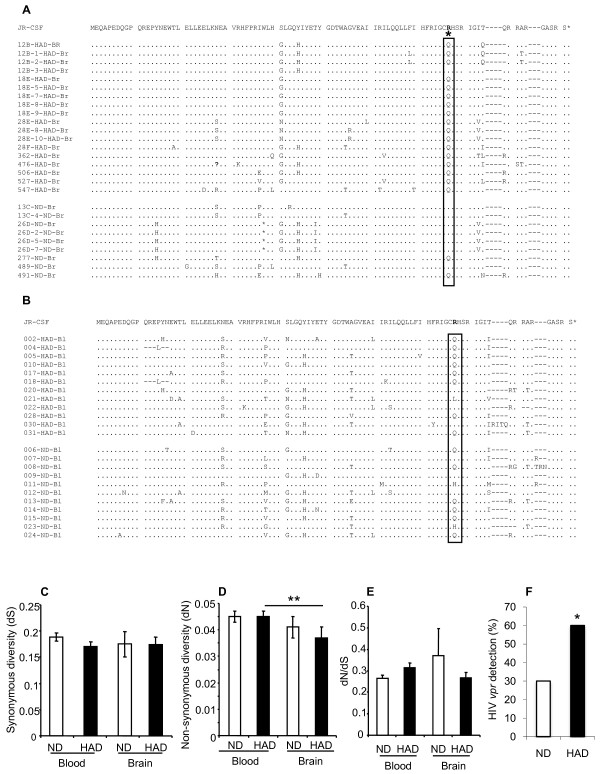
**Brain- and blood-derived Vpr sequences**. (A) Brain-derived sequences exhibited diversity in both the HAD and ND groups but a mutation at position 77 significantly distinguished the clinical groups with a Q predominating in the HAD group and an R being most evident in the ND group. (B) Blood-derived sequences also demonstrated molecular heterogeneity in both groups but there were no residues that distinguished the clinical groups. (C) The frequency of within-groups synonymous mutations was similar among all sequences from all clinical groups. (D) The frequency of within-group non-synonymous mutations was lower in the brain-derived HAD sequences compared with the blood-derived HAD sequences. (E) Conversely, the ratios of within-group non-synonymous to synonymous mutations did not differ within the clinical groups. (F) The frequency of detecting *vpr *sequences in brain was significantly higher in the HAD group compared with the ND groups (A, B, F: Mann-Whitney *U *test; C-D: ANOVA, Bonferroni *post hoc *test; *p < 0.05).

Previous studies indicated both Vpr-encoding transcripts and proteins were present in the brains of HIV-infected persons [20,33], chiefly in cells of monocytoid lineage in keeping with other studies of HIV neurotropism [34,35]. To extend these analyses, full length *vpr *sequences were amplified from subcortical frontal white matter and PBMCs from HAD and ND patients. Alignment of the predicted amino acid sequences showed that there was substantial heterogeneity throughout the brain-derived sequences among both HAD and ND patients using the HIV-1 JR-CSF Vpr sequence as a reference. However, at amino acid residue 77, there was a significant sequence dichotomy in that a glutamate (Q) predominated in HAD clones (17/18) but at the same position, an arginine (R) was chiefly present in ND clones (7/9) (Figure 1A). To verify this observation, we analyzed blood-derived sequences from HAD and ND AIDS patients, which showed molecular diversity at multiple positions in both the HAD and ND groups but the amino acid changes distinguishing HAD and ND in brain were not evident (Figure 1B). The nature of the molecular diversity in *vpr *was investigated further by examining the diversity of synonymous mutations within clinical groups, which did not differ within blood- or brain-derived sequences from each group (Figure 1C). The frequencies of non-synonymous mutations was significantly lower within the HAD brain-derived sequences compared with the HAD blood-derived sequences (Figure 1D). Conversely, the dN/dS rates did not differ among blood- and brain-derived sequences (Figure 1E). Complementing the observation of a lower non-synonymous rate in HAD brain-versus blood-derived sequences, the numbers of amino acid differences were also significantly lower in the HAD brain-derived sequences than in HAD blood-derived sequences (data not shown). However, the frequency of detection of *vpr *transcripts in brain was significantly higher among HAD patients (59%) compared with ND patients (31%) (Figure 1F). In contrast, *vpr *transcripts were detected in all blood-derived samples examined, regardless of clinical diagnosis. These observations highlighted a distinct mutation which distinguished HAD from ND brain-derived *vpr *sequences together with greater rates of *vpr *transcript detection in HAD brains.

### Intracellular actions of Vpr 77R and 77Q

**Figure 2:**
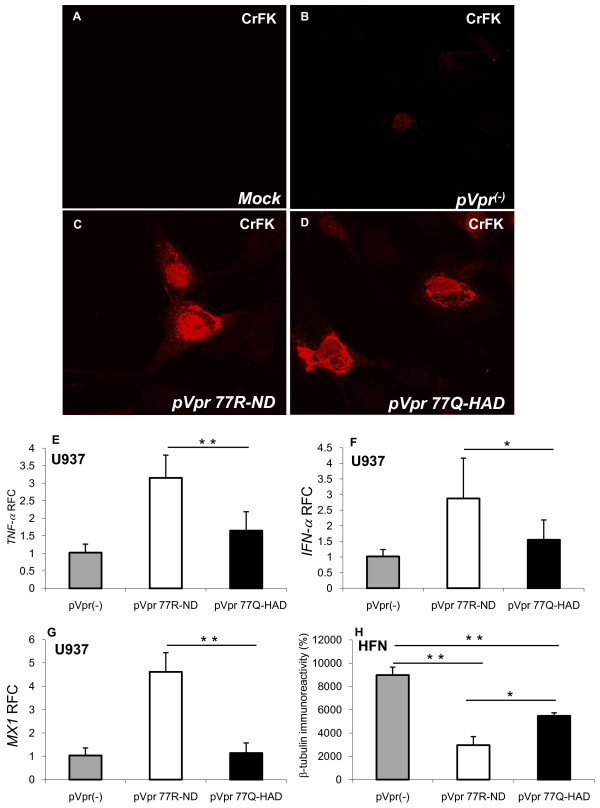
**Expression and intracellular actions of Vpr 77Q and 77R**. (A) Mock-transfected CrFK cells exhibited no Vpr immunoreactivity; (B) A non-expressing Vpr plasmid (p*Vpr*^***(-)***^) also show weakly Vpr immunoreactivity in transfected CrFK cells; (C) and (D) Vpr immunoreactivity was readily detected in the cytoplasm and nuclei of CrFK cells transfected with (C) p*Vpr77R-ND *and (D) p*Vpr77Q-HAD; *(E) p*Vpr77R-ND *transfection of U937 cells caused an induction of *TNF-α/vpr *transcript abundance relative to p*Vpr*^***(-)***^; (F) likewise, p*Vpr77R-ND *activated *IFN-α/vpr *transcription in U937 cells; (G) p*Vpr77R-ND *also induced expression of *MX1/vpr*; (H) Supernatants from both p*Vpr77Q-HAD *and p*Vpr77R-ND *transfected U937 cells were neurotoxic to human fetal neurons (HFN), as evidenced by reduced β-tubulin immunoreactivity, although the supernatants from the p*Vpr77R-ND *transfected U937 cells were more cytotoxic. Original magnification 600×. Real time PCR data was normalized against the matched *Vpr *mRNA levels. Experiments were carried out in triplicate at least two times (E-G, Dunnett test, relative to control; *p < 0.05, **p < 0.01).

Diversity at amino acid position 77 has been previously recognized in blood-derived samples from HIV/AIDS although the associated effects of this mutation in the nervous system were uncertain [27,29,36]. To determine the actions of each amino acid at position 77 on immune activation and the consequent effects on neuronal viability, the full length *vpr *allele was cloned and thereafter mutated at position 77, generating 77Q-(p*Vpr77Q-HAD*) or 77R (p*Vpr77R-ND*)-containing vectors. To ensure expression of the Vpr protein, Vpr immunoreactivity was analyzed following transfection of cultured CrFK cells with 77R- or 77Q-containing *vpr *vectors, together with a non-expressing vector (p*Vpr*^*(-)*^) and mock transfection (Figure 2). In the non-expressing vector (p*Vpr*^*(-)*^), the *Vpr *start codon "ATG" was substituted to "ACG". As expected, Vpr immunoreactivity was not detectable in the mock (Figure 2A) and was minimally detectable in the p*Vpr*^*(-)*^-transfected cells (Figure 2B) [37]. However, Vpr immunoreactivity was abundant in the cytoplasm and nuclei of cells transfected with the p*Vpr77R-ND *(Figure 2C) and p*Vpr77Q-HAD *(Figure 2D) vectors, confirming the expression of Vpr by 77R and 77Q vectors.

Vpr has been reported to exert both immune and cytotoxic effects depending on the model [20,25,38-41]. To assess the effects of each *vpr*-containing vector, immune gene expression was measured in electroporation-transfected myeloid (U937) cells, which revealed that p*Vpr77R-ND *induced *TNF-α *significantly more than p*Vpr77Q-HAD *and p*Vpr*^*(-) *^(Figure 2E). Likewise, p*Vpr77R-ND *also significantly activated *IFN-α *(Figure 2F) and *MX1 *(Figure 2G) transcriptional activity in monocytoid cells. These studies were extended by assessing the neurotoxic effects of supernatants from transfected cells applied to human fetal neurons (Figure 2H), which demonstrated that supernatants derived from p*Vpr77R-ND*- and p*Vpr77Q-HAD-*transfected myeloid cells caused significant reductions in neuronal viability, measured by β-tubulin immunoreactivity in human fetal neurons compared with supernatants from the p*Vpr*^*(-)*^-transfected cells. However, the supernatants from the p*Vpr77R-ND-*transfected myeloid cells were significantly more neurotoxic in this assay. These studies highlighted Vpr's capacity to induce variable neuroimmune responses, depending on the individual Vpr allele but also underlined an association between immune response and related neurotoxicity with the supernatants from p*Vpr77R-ND*-transfected cells showing the greatest neurotoxicity.

### Transduction of glial cells with viruses expressing Vpr mutants

**Figure 3:**
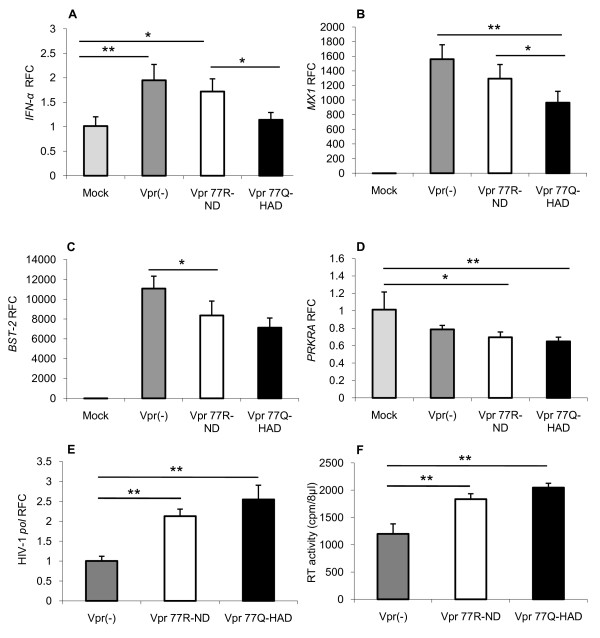
**Human astrocyte transfection with HIV-1 Vpr mutant viruses**. (A) Transduced astrocytes showed that pseudotyped virus (pv) expressing Vpr77Q induced the least *IFN-α *expression. Similarly, the Vpr77Q virus induced (B) *MX1 *and (C) *BST-2 *transcript levels were lowest in the Vpr77Q virus-transduced astrocytes; while (D) *PRKRA *was consistently reduced by all HIV-1 vectors. (E) In contrast to the host gene expression observed in A, B and C, HIV-1 *pol *were highest in the Vpr77Q virus-transduced astrocytes, which was complemented by a similar profile in RT activity in matched supernatants (F). Experiments were carried out in triplicate at least two times (A-F, Dunnett test, relative to control; *p < 0.05, **p < 0.01).

In addition to studying the actions of Vpr in isolation, its effects were examined in the context of whole virus expression in which viruses encoding Vpr 77R, 77Q or null were constructed. All of the viruses induced *IFN-α *expression following transduction of human astrocytes, although there was least *IFN-α *activation in the Vpr 77Q-encoding virus-transfected cells (Figure 3A). Likewise, all virus-transduced astrocytes displayed induction of *MX1 *(Figure 3B) and *BST-2 *(Figure 3C) but again lowest levels were observed in the Vpr 77Q-encoding virus-transduced cells for both host genes. Conversely, all of the virus-transduced cells exhibited reduced *PRKRA *expression relative to the mock-transduced astrocytes (Figure 3D). HIV-1 *pol *mRNA levels were detected in all transduced cells but were highest in cells transfected with the Vpr 77Q-encoding virus (Figure 3E), which was complemented by a similar profile in RT activity in matched supernatants (Figure 3F). These findings suggested an inverse relationship between viral gene expression and specific host immune responses, depending on both the presence and sequence of Vpr.

### Vpr peptides (aa 70-96) activate neuronal calcium fluxes

**Figure 4:**
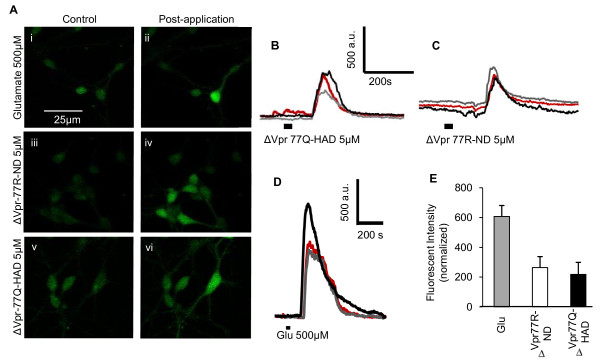
**Vpr peptides (aa 70-96) increase cytosolic Ca^2+ ^fluxes in human neurons**. (A) Confocal imaging of Fluo-4-labeled human neurons before and after application of glutamate (i and ii), ΔVpr77R-ND (iii and iv) and ΔVpr77Q-HAD (v and vi) showing an increase in calcium flux for all exposures. (B-D) Representative traces showing time courses of calcium fluxes in human neurons after exposure to glutamate (B), ΔVpr77Q-HAD (C) and ΔVpr77R-ND (D) peptides. The thick black line represents the duration during which glutamate or the peptides were applied. (E) Graphic representation of the relative fluorescent intensity ΔVpr77R-ND and ΔVpr77Q-HAD relative to glutamate response, showing similar levels of fluorescence induction for both peptides (Student *t *test). Original magnification 200×.

While Vpr is expressed within cells as part of viral transport to the nucleus as well as viral assembly [42-45], it is also secreted into cerebrospinal fluid and plasma and acts at the neuronal membrane to influence neuronal function and survival [22,46]. It has been previously shown that a C-terminal domain of the Vpr protein (amino acids 70-96) has a critical role in Vpr-mediated cytotoxic effects [47]. Given that the R77Q mutation was located within this domain of the protein, we investigated the effects of the amino acid 77 mutation using 70-96 Vpr peptides, containing either Vpr77Q (ΔVpr77Q-HAD) or Vpr77R (ΔVpr77R-ND). Previous reports indicate that Vpr is capable of reducing neuronal viability by inducing apoptosis as well as perturbing the cell cycle machinery [20,47-49]. However, its effects on intracellular calcium fluxes in neurons are less certain. Vpr peptides' actions on neuronal cytosolic calcium mobilization were assessed by confocal microscopy in Fluor-4 prior-loaded human neurons. Glutamate (500 μM), which was used a positive control, activated robust responses in terms of changes in intracellular calcium concentrations [Ca^2+^]_i _(Figure 4A) but in addition, both ΔVpr77R-ND (n = 30) and ΔVpr77Q-HAD (5.0 μM) (n = 19) also activated calcium responses in human neurons. The temporal profiles of Vpr peptides' actions were similar to glutamate, albeit at lower signal amplitudes (Figure 4B-E). This observation was confirmed by graphic analysis, which showed that the Vpr peptides caused smaller changes in [Ca^2+^]_i_, compared with glutamate exposure to neurons (Figure 4E). Thus, in contrast to the assays described above, amino acids Q or R at position 77 within Vpr modulated calcium responses similarly in neurons.

### Mutant Vpr peptides (aa 70-96) show differential effects on host immune responses

**Figure 5:**
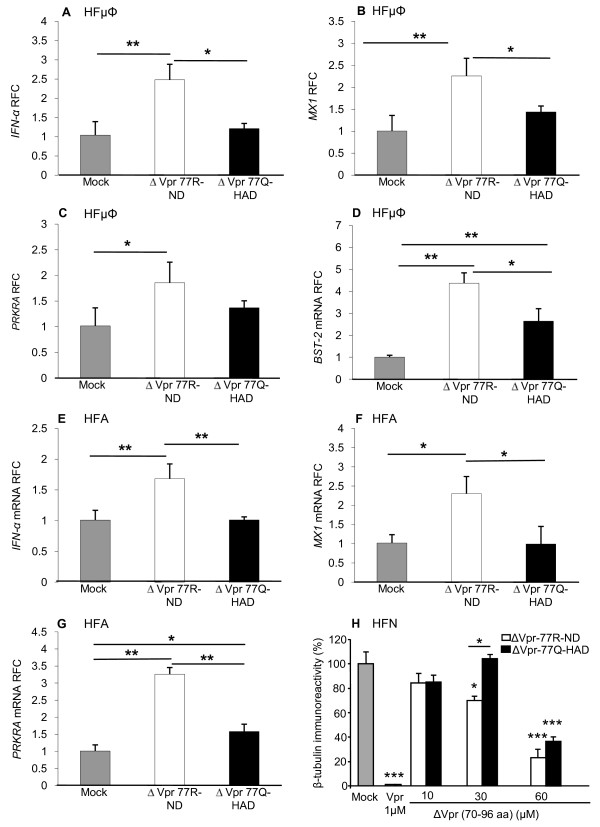
**Vpr peptides (aa 70-96) exert neuroimmune and neurotoxic effects**. Exposure of ΔVpr77Q-HAD (5.0 μM) or ΔVpr77R-ND (5.0 μM) to human microglia resulted in ΔVpr77R-ND-mediated induction of (A) *IFN-α*, (B) *MX1*, (C) *PRKRA *and (D) BST-2 transcripts. Similarly, human astrocytes exposed to the same peptides showed induction of (E) *IFN-α*, (F) *MX1 *and (G) *PRKRA *expression. (H) Exposure of ΔVpr77R-ND to human neurons caused a concentration-dependent (10.0-60.0 μM) reduction in β-tubulin immunoreactivity while ΔVpr77Q-HAD showed less neurotoxicity. Full length Vpr (1.0 μM) was also highly neurotoxic. Experiments were carried out in triplicate at least two times (A-D, Dunnett test, relative to control; *p < 0.05, **p < 0.01).

Since microglia and astrocytes represent the principal innate immune cells within the brain, the actions of soluble Vpr on their function were highly relevant to the present experiments. Human fetal microglia (HF μΦ) were exposed to Vpr peptides revealing that the ΔVpr77R-ND peptide activated greater *IFN-α *(Figure 5A), *MX1 *(Figure 5B), *PRKRA *(Figure 5C) and *BST-2 *(Figure 5D) expression compared with ΔVpr77Q-HAD- or mock-exposed microglia. Likewise, human fetal astrocytes (HFA) exposed to the ΔVpr77R-ND peptide displayed the highest induction of *IFN-α *(Figure 5E), *MX1 *(Figure 5F) and *PRKRA *(Figure 5G). Both ΔVpr peptides did not activate expression of *IL-1β *or *TNF-α *in both primary human cell types (data not shown). ΔVpr peptides were also applied to human fetal neurons (HFN) showing ΔVpr77R-ND (30.0 μM) was neurotoxic while ΔVpr77Q-HAD (30.0 μM) did not differ from the mock-exposed cultures (Figure 5H). Both ΔVpr77R-ND and ΔVpr77Q-HAD (60.0 μM) significantly reduced β-tubulin immunoreactivity but again ΔVpr77R-ND was more neurotoxic at this concentration. Of note, the full length (amino acids 1-96) Vpr (1.0 μM) was substantially more neurotoxic than both Vpr peptides, emphasizing the importance of the full length Vpr molecule for mediating Vpr's neurovirulent properties.

### *In vivo *actions of Vpr and derived peptides

**Figure 6:**
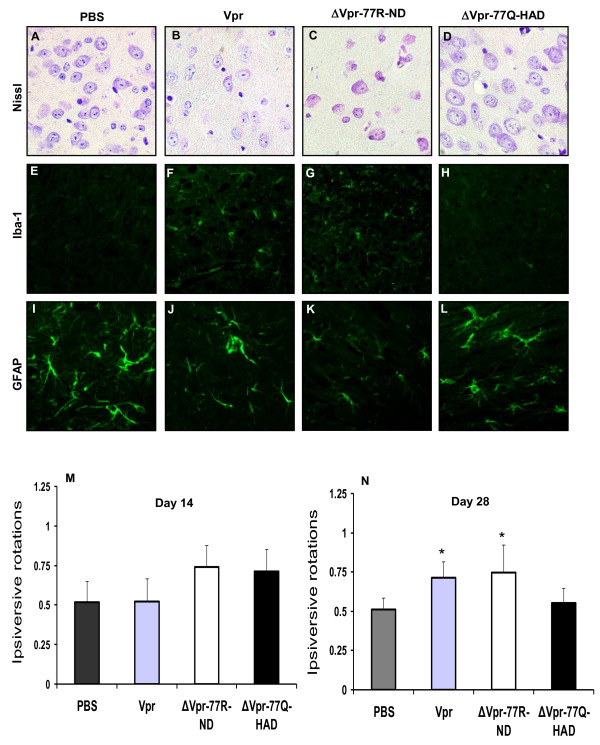
**Implanted Vpr and Vpr-derived peptides exert differential effects *in vivo***. Nissl-stain preparations of ipsilateral basal ganglia (A-D) displayed more neurons in animals implanted with PBS (A) or ΔVpr77Q-HAD (D) compared with full length Vpr (B) or ΔVpr77R-ND (C) implanted animals. Iba-1 immunoreactivity was minimally detected in animals implanted with PBS (E) or ΔVpr77Q-HAD (H) compared with full length Vpr (F) or ΔVpr77R-ND (G) implanted animals, which showed numerous microglia. GFAP immunoreactivity was increased in animals implanted with PBS (I) or ΔVpr77Q-HAD (L) compared with full length Vpr (J) or ΔVpr77R-ND (K) implanted animals. Ipsiversive rotary behaviour fraction (relative to the total number of rotations) did not differ significantly between groups at day 14 post-implant (M) but at day 28 both full length and ΔVpr77R-ND (N) implanted animals showed great ipsiversive rotations. The number of animals used in each experimental group is as follows: PBS group: n = 3; Vpr group: n = 3; ΔVpr-77R-ND group: n = 4; ΔVpr-77Q-HAD group: n = 4 (M-N, Dunnett test, relative to control; *p < 0.05). Original magnification 400×.

Vpr causes neurodegeneration and neurobehavioral deficits in transgenic mice selectively expressing Vpr in microglia [20-22,33]. However, the actions of soluble Vpr proteins or peptides expressed focally in the brain were unknown. Full length Vpr (amino acids 1-96), ΔVpr77R-ND, ΔVpr77Q-HAD or PBS were stereotactically implanted into the striatum of mice and subsequent neuropathological and neurobehavioral studies were performed. Neuropathological studies of the basal ganglia revealed that numerous neurons, identified by their prominent nuclei and nucleoli in Nissl-stained preparations, were present in the basal ganglia of PBS-implanted animals (Figure 6A) but in contrast there were a reduced number of neurons in animal implanted with the full length Vpr- (Figure 6B) and ΔVpr77R-ND- (Figure 6C). No differences in neuronal abundance from the PBS-implanted animals were observed in the ΔVpr77Q-HAD-implanted animals (Figure 6D). Minimal Iba-1 immunoreactivity was evident in the basal ganglia of PBS-implanted animals (Figure 6E) while the numbers of Iba-1 immunopositive microglia were increased in the full length Vpr- (Figure 6F) and ΔVpr77R-ND- (Figure 6G) implanted animals, reflecting a glial response to cellular injury. Iba-1 immunoreactivity did not differ between the PBS-implanted animals and the ΔVpr77Q-HAD-implanted animals (Figure 6H). GFAP immunoreactivity was readily detected in astrocytes of the PBS-implanted animals (Figure 6I) but was diminished in the full length Vpr- (Figure 6J) and ΔVpr77R-ND- (Figure 6K) implanted animals while GFAP immunoreactivity in the ΔVpr77Q-HAD-implanted animals (Figure 6L) was similar to the PBS-implanted control animals.

To define the neurobehavioral correlates accompanying the neuropathological studies described above, ipsiversive rotary behavior was recorded at days 7, 14, and 28 post-implantation. These studies disclosed that at days 7 (data not shown) and 14, experimental groups displayed similar levels of ipsiversive rotary behavior (Figure 6M). However, at day 28 post-implantation, both full length Vpr and each ΔVpr77R-ND caused significantly increased rotary behavior compared with PBS-implanted animals (Figure 6N). Thus the latter findings supported the present *in vitro *and neuropathological findings in that Vpr containing 77R, as a peptide or full length protein, was more neurovirulent compared with the 77Q peptide or controls.

## Discussion

In the present studies, mutations at amino acid position 77 were discovered within brain-derived HIV-1 Vpr sequences, which distinguished HIV/AIDS patients with (77Q) and without (77R) HIV-associated dementia. Remarkably, these mutations varied in their ability to induce innate immune responses depending on the specific mutation, which were also associated with their neurodegenerative actions. Moreover, ΔVpr peptides (amino acids 70-96), containing the variable amino acid 77 residue, exerted both immunogenic and neurotoxic actions *in vitro *and *in vivo *but the ensuing outcomes were influenced by the specific mutation present at position 77 within the peptide. Although the 77R mutation induced greater antiviral innate immunity and increased neurotoxicity, the 77Q mutation was associated with higher frequency of detection in human brain and replicated at similar levels to the virus containing the 77R mutation in glial cells. These findings highlighted the complexity of events influenced by HIV-1 molecular diversity, together with the additive effects of viral molecular heterogeneity on host responses and viral replication in the development of neurovirulence.

Vpr is expressed by the HIV genome later in the viral life cycle but it appears essential for macrophage infection and perhaps microglia tropism. Vpr also mediates apoptosis in multiple cells types, possibly through influencing G2 phase of the cell cycle [15,50-54]. Previous reports indicate that Vpr exhibits neurovirulent properties including alterations in neuronal excitability and ensuing death *in vitro *as well as synaptic retraction *in vivo*, accompanied by neurobehavioral abnormalities [20]. As in previous studies, Vpr-derived peptides were neurotoxic [47,55-57] while for full length and the derived peptides, innate immune activation was largely limited to antiviral responses (IFN-α and BST-2 induction) with limited concurrent induction of proinflammatory cytokines (IL-1β, TNF-α). This latter observation highlights Vpr's neurodegenerative aspects, which are not linked *per se *to pro-inflammatory mechanisms in the nervous system. Regulation of innate immune responses is a pivotal determinant of progression to AIDS but also influences the development of HIV-induced brain disease [58-60]. Type I interferons, interferon (IFN)-α and -β, exert antiviral effects through multiple pathways including regulation of the expression of several downstream genes including *MX*, *PRKRA*, and *BST-2*, all with potential antiviral activities [61-63]. MX proteins are a group of dynamin-like large guanosine triphosphatases (GTPases) enzymes. Some MX GTPases have been shown to exert antiviral effects against a wide range of RNA and some DNA viruses [64]. PRKRA is an interferon-inducible protein kinase, also known as Protein kinase R (PKR)-activating protein, which is involved in PKR-mediated antiviral effects [65]. Likewise, bone marrow stromal cell antigen 2 (BST-2), also termed tetherin, has also been shown to be an IFN-regulated restriction factor for HIV-1 [63,66]. While neuroinflammation is a cardinal feature of HAD, antiviral responses including induction of IFN-α, MX-1, PRKRA or BST-2 await clarification of their expression in HAD, although several studies indicate the IFN-α might be increased in the brains of HIV/AIDS patients [67-69].

Molecular diversity, as well as specific mutations within the HIV-1 genome, has been associated with HIV-induced neurological disease [32,70,71]. In particular, increased diversity within brain-derived HIV-1 envelope sequences from HAD patients is a common finding in several studies [71,72]. Specific mutations and/or motifs within HIV-1 gp120 have also been associated with HAD [73]. Differential sequence diversity within brain-derived Tat and Nef sequences appear to discriminate between HIV/AIDS patients with and without HAD [74-76]. It was shown that astrocytes would harbor provirus only [77], therefore viral genomic RNA used as template for RT-PCR to amplify vpr gene in this study should be derived from perivascular macrophages or microglia. Herein, amino acid position 77 within Vpr distinguished the two clinical groups, 77Q and 77R in HAD and ND AIDS patients, respectively. Our finding that brain-, but not blood-derived, sequences distinguished HAD from ND AIDS patients implies the motif at position 77 might reflect mutagenesis of the virus within the brain. The 77Q mutation has been associated with sustained non-progression of HIV infection [27,29], while in the present study the same mutation was associated with HAD. Protein sequence alignment of HIV-1 Vpr from 4 HIV-1 B clade strains revealed that prototypic brain-derived viruses, YU2 and JRFL, from patients with HAD exhibited 77Q while non-brain-derived strains (JR-CSF and NL4-3) show 77R. This comparison suggests that the change from 77R to 77Q might be important for both neurotropism and perhaps neurovirulence. Similar to previous studies, the 77Q motif also exerted less cytotoxic effects and minimal induction of anti-viral immune responses *in vitro*, suggesting this same mutation also diminished cytopathogenicity [28]. It is widely assumed that HAD represents a state of increased HIV-1 neurovirulence, recapitulating animal studies in which a specific virus causes neurovirulence [78,79]. Thus, the present studies raise a dichotomy regarding Vpr's role in neurovirulence: although the 77Q motif was more frequently detected in brain-derived sequences from HAD patients, the same mutation caused less neurotoxicity and a muted antiviral immune response. However, the likelihood of detecting *vpr *sequences in brain was significantly higher in HAD (Figure 1F) and the viruses encoding Vpr 77Q or 77R replicated similarly in glial cells (Figure 3E and 3F). Several potential explanations underlie these findings: (a) the 77Q mutation with Vpr permits HIV-1 to persist and replicate in the brain by restricting the neuroimmune antiviral response(s) and Vpr's direct neurotoxic effects, thereby augmenting the virus' fitness and replicative capacity, as evidenced by its increased detection in HAD brains and the apparent inability to induce BST-2; (b) the Vpr 77Q mutation might be an associated or compensatory mutation, which modulates viral replication but is enhanced by other neurovirulence-conferring mutations occurring elsewhere in the HIV genome, thereby preventing the virus from overwhelming the host; (c) the 77Q confers some as yet unrecognized property on the virus in terms of its neurovirulence, perhaps through its putative effects on phosphorylation of the nearby 79S residue within Vpr [80,81]. Regardless of what pathogenic mechanism is mediated by the 77Q or -R motifs, the brain is likely an "evolutionary cul-de-sac" for the virus because HIV-induced brain disease predicts worsened survival with or without combination antiretroviral therapy [82,83]. The present findings raise a fundamental issue regarding the relationship between virus-mediated neurovirulence and neurological outcomes, suggesting that HAD might be a state of failing neuroimmunity.

The present observations highlight an important aspect of HIV disease progression in a cohort of AIDS patients regarding the role of a comparatively unstudied viral protein found in the brain. However, these studies require verification in a larger cohort and perhaps in patients infected with different HIV-1 clades. The use of the present animal model could be extended by comparing transgenic animals containing each amino acid 77 within the expressed Vpr. Herein GFAP immunoreactivity was diminished in the basal ganglia in brains of mice implanted with the ΔVpr-77R-ND peptide, whereas as it was not significantly altered in the basal ganglia of mice receiving ΔVpr-77Q-HAD peptide (Figure 6I-L), indicating the wild-type but not the mutant Vpr peptide exerted a cytotoxic effect on astrocytes. Moreover, mice receiving full-length Vpr injection also showed a reduction in GFAP immunoreactivity, indicative of astrocyte injury/death, which was consistent with recent observations in the brains of Vpr-transgenic animals [84]. Future studies of cerebrospinal fluid (CSF)-derived Vpr alleles might also be a fruitful approach in terms of understanding pathogenesis as well as diagnostic importance, given the availability of CSF early in the disease course.

## Conclusions

These observations suggest that the 77R mutation within Vpr exerts greater effects on host cell immunity and survival than 77Q, thereby limiting viral expression and perhaps persistence in the brain. However these findings also indicate that HIV-mediated neurovirulence reflects the virus' overall capacity to curb antiviral immune responses through viral mutagenesis coupled with preserving its replicative properties.

## Methods

### Human brain and blood samples for RNA isolation, PCR and sequencing

Genomic RNA was isolated from frontal white matter of brain tissue and peripheral blood mononuclear cells (PBMCs), which were obtained from AIDS-defined HIV-1 seropositive persons who were non-demented (ND) or diagnosed pre-mortem with HAD, using TRIzol reagent (Gibco), as previously reported [85,86]. According to the manufacturer's protocol, total RNA was isolated, dissolved in diethylpyrocarbonate (DEPC)-treated water and used for the synthesis of cDNA. The HIV-1 *vpr *gene was amplified from cDNA using a nested PCR protocol: initial denature step of 2 min at 94°C, followed by 35 cycles of 30 S at 94°C, 30 S at 52°C, 1 min at 68°C, with a final extension step of 7 min at 72°C. The forward and reverse primers used were as follows: first round forward primer 5'-CAAGCAGGACATAACAAGGTA G; first round reverse primer 5'-TGGCAATG AAAGCAACACT; second round forward primer 5'-CATCTAGAGCAGAGGACAGATGGAACAAG and second round reverse primer 5'-CTAG GCCTTCTAGGATCTACTGGCTCC. PCR fragments corresponding to the amplified *vpr *gene were isolated from agarose gel using the QiaQuick gel extraction kit (Qiagen), the incomplete fragment ends were filled in with Klenow, phosphorylated using T4 polynucleotide kinase and cloned into the pSL1180 vector (Amersham Biosciences Inc). All reagents were obtained from New England BioLabs Ltd and used following the manufacturer's specifications. PCR fragments or multiple clones of the cloned PCR fragments were sequenced in both directions using the second round PCR forward and reverse primers, as previously reported [72,76,85]. DNA sequences were determined by automated sequencing on an ABI 370 sequencer (Applied Biosystems, Streetsville, ON) using the manufacturer's protocols and reagents. The sequences obtained from cloned and PCR fragments were aligned using the BioEdit sequence alignment software (Ibis Biosciences, Carlsbad, CA) and used to derive a consensus sequence for each patient group.

### Construction of *vpr *clones

To clone 96 amino acid HIV-1 *vpr *gene (derived from HIV-1 NL43), forward primer PHN96-1F 5'-GGGCCCGGGATCCACCGGTCGCCACCATGGAACAAGCCCCAGAAGACC, containing *Bam*H I restriction site, and reverse primer PHN96-1RM 5'-GGCGGATACCCGCG GCCGCCTAGGATCTACTGGCTCCATTTC, containing *Not *I restriction sites, were utilized with template of HIV NL4-3 plasmid cDNA to amplified the *vpr *fragment by PCR. The generated fragment was subsequently digested with *Bam*H I and *Not *I and ligated into a pEGFP-N1 plasmid vector with same restriction enzymes digestion, in which the *green fluorescent protein *gene was removed. Similarly, a *vpr *start codon knockout clone was constructed by replacing above primer PHN96-F with PHN96-1FK5'-GGGCCCGGGATCCACCGGTCGCCA CCACGGAACAAGCCCCAGAAGACC, in which ATG was substituted to ACG [37]. To construct the "R77Q" modified HIV *vpr *(NL43D), primer pairs of PHN96-1F (shown above)/PHN96-3R 5'-TCTGCTATGTTGACACCCAATTCTG and PHN96-1RM (shown above)/PHN96-3F 5'-GTGTCAACATAGCAGAATAGGC were used to generate two overlapping fragments of the *vpr *gene with template of HIV NL4-3 plasmid for the first PCR. The two generated fragments were fused together in a subsequent extension reaction and amplified by secondary PCR with outside primers PHN96-1F and PHN96-1RM (shown above) and thus generate an entire "R77Q" modified *vpr *gene fragment flanked with *Bam*H I and *Not *I restriction site. The entire *vpr *gene fragment was subsequently cloned into above described GFP vector generating NL4-3. The two *vpr *constructs, 77R-ND and 77Q-HAD were re-sequenced to ensure that only the *vpr *gene sequences of interest were present.

### HIV molecular clones' construction

To evaluate the effects of Vpr mutations on human fetal astrocytes, HIV-1 envelope-defective molecular clones were constructed for transfection. The construction of HIV-1 envelope-defective proviral plasmids, including HxBRUR-/Env-, HxBRUR+/Env-, and HxBRUR+/Env-/*Vpr*(R77Q) was previously described [27,87,88].

### Transfection and immunofluorescence detection

To test expression of cloned HIV-1 Vpr protein, transfection of the cloned HIV-1 *vpr *plasmid was performed in Crandle feline kidney (CrFK) cells (ATCC) and expression of the Vpr protein within CrFK cells was subsequently analyzed by immunofluorescent staining. 1.5 × 10^5 ^CrFK cells were cultured on a sterile cover slip in MEM medium supplemented with 10% fetal bovine serum, penicillin (100 U/ml), and streptomycin (100 μg/ml) by incubation at 37 degrees Celsius with 5% CO_2 _for 24 hours to achieve >90% confluency. 1.5 μg of *vpr *plasmid DNA and 5 μl of Lipofectamine 2000 (Invitrogen) in 500 μl of Opti-MEM medium (Gibco) were transfected into each well according to the manufacturer's protocols. For immunofluorescent staining, supernatants of the overnight transfected CrFK cells were replaced with 1 ml 2% (pH7.4) PBS-buffered paraformaldehyde and incubated for 20 min at room temperature (RT). Cells were washed with PBS 2 × 5 min followed by incubation with blocking buffer (PBS/0.2% Triton/10% normal goat serum and 2% BSA) for 1 hour at room temperature. 100 μl diluted rabbit anti-vpr primary antibody (1:100 dilution with PBS/0.2% Triton/5% normal goat serum and 1% BSA) was added on each coverslip and incubated for 2 hours at RT. A shaking wash step with PBS (0.1% Tween20) at RT for 3 × 10 min was followed incubation with the primary antibody. Subsequently, 100 μl diluted goat anti-rabbit secondary antibody (Cy3 Conjugated) (1:1000 dilution PBS/0.2% Triton/5% normal goat serum and 1% BSA) was added on each coverslip and incubated for 1 hours at RT. Shaking washes with PBS (0.1% Tween20) at RT for 4 × 15 min were performed and the coverslips were mounted with Gelvatol on glass slides for confocal laser-scanning microscopy analysis as described previously [20].

### Human myeloid cell (U937) transfection by electroporation

10 μg of cloned *vpr *plasmid was added to 5 × 10^6 ^U937 cells/reaction which then were re-suspended in 250 μl of room temperature RPMI medium (without FBS and P/S). The mixture was then transferred to a 0.4-cm gap cuvette for electroporation. Electroporation was performed at 242 voltage and 975 microfarads for ~30 msec with a Gene Pulser II electroporator (Bio-Rad). The electroporation-transfected U937 cells were cultured in 12 well plates, adding fresh RPMI medium (1 ml/well) containing 10% fetal bovine serum, penicillin (100 U/ml), and streptomycin (100 μg/ml), followed by incubation at 37°C in 5% CO_2 _for 24 hours. At 24 hours post-transfection, culture supernatants were collected for neurotoxicity assays and total cellular RNA was isolated using the TRIzol reagent (Gibco).

### Human fetal neuron, astrocyte and microglia cell cultures

Human neuronal cultures were prepared from 15-19 week aborted fetal brains obtained with consent (approved by the University of Alberta Ethics Committee), as previously described [86]. Briefly, fetal brain tissues were dissected, meninges were removed, and a single cell suspension was prepared by trituration through serological pipettes, followed by digestion for 30 min with 0.25% trypsin (Life Technologies, Burlington, ON, Canada) and 0.2 mg/ml DNase I (Roche Diagnostics, Mannheim, Germany) and passage through a 70-μm cell strainer (BD Biosciences, Mississauga, ON, Canada). Cells were washed 2 times with fresh medium and plated in T-75 flasks coated with poly-L-ornithine (Sigma-Aldrich, Oakville, ON, Canada) at 6-8 × 10^7 ^cells/flask and medium for growing human fetal neuron (HFN), astrocyte and microglia cells was subsequently added (named HFN medium), which was MEM supplemented with 10% FBS (Life Technologies), 2 mM L-glutamine (Life Technologies), 1 mM sodium pyruvate (Life Technologies), 1 × MEM nonessential amino acids (Life Technologies), 0.1% dextrose (Sigma-Aldrich), 100 U/ml Penicillin (Life Technologies), 100 μg/ml streptomycin (Life Technologies), 0.5 μg/ml amphotericin B (Life Technologies), and 20 μg/ml gentamicin (Life Technologies). Specifically, for neuronal cultures, 25 μM cytosine arabinoside (Sigma-Aldrich) were additionally supplemented to prevent astrocyte growth. Astrocyte cultures, without cytosine arabinoside, were passaged once a week and in 4-6 weeks the neurons were eliminated; the remaining astrocytes were ready for HIV transfection or infection. For microglial cell cultures, suspended microglial cells collected by centrifugation at 1200 rpm for 10 min at one week after cultures were established. The collected microglia cells were grow in a new plate with the above medium (without cytosine arabinoside) and ready for HIV transfection or infection in two days.

### Neuronal toxicity assay

Neurotoxicity assays were performed by methods described previously [20]. Briefly, human fetal neurons described above were cultured in 96-well flat bottom plate with supernatants derived from transfected U937 cells for 48 hours. Cells were then fixed in 4% formalin, washed in PBS containing 0.1% Triton-X, blocked for 90 min with LI-COR Odyssey Blocking Buffer (LI-COR, Lincoln, NE). After removal of the blocking reagent, the cells were incubated overnight at 4°C with monoclonal mouse anti-β-tubulin (1:1000 dilution; Sigma-Aldrich). After primary antibody application, the cells were washed in PBS containing 0.1% Tween-20 and incubated with goat anti-mouse Alexa Flour 680 (1:200 dilution; Invitrogen) secondary antibody. All antibody dilutions were made with LI-COR Odyssey Blocking Buffer. After removal of the secondary antibodies, the cells were washed in PBS/0.1% Tween 20 and left to dry in the dark before quantification of β-tubulin immunoreactivity using the Odyssey Infrared Imaging System (LI-COR).

### Human astrocyte (HFA) transfection

The above prepared HFAs were subsequently seeded into 12 well plates at 2 × 10^**5 **^cells/well and 1 × 10^**4 **^cells/well, respectively, followed by incubation at 37 degrees Celsius with 5% CO_2 _for 24 hours. For each transfection reaction, 2 μg of proviral HIV-1 plasmid (HxBRUR+/ENV-, HxBRUR-/ENV- or HxBRUR+/ENV-/*Vpr*(R77Q)) were mixed with 1 μl plus reagent (Invitrogen) for 10 minutes. Then 5.5 μl of Lipofectamine LTX (Invitrogen) was added to the mixture and incubated for 30 minutes followed by transfection to each well (with 1 ml fresh HFN medium) according to the manufacturer's protocols. At 6 hours post-transfection, transfection medium was removed and 1 ml/well fresh HFN medium was added. At 48 hours post-transfection, culture supernatants containing pseudotyped HIV-1 were collected for viral reverse transcriptase (RT) activity assay and total cellular RNA was isolated from the cultured primary HFAs with using RNeasy Mini Kit (Qiagen) after lysis with TRIzol (Invitrogen) using manufacturer's guidelines. The isolated RNA was used for real time RT-PCR.

### Reverse transcriptase (RT) assay

RT activity in culture supernatants was assayed as previously described [89]. Briefly, 8 μl of culture supernatant was incubated with 32 μl of reagent buffer containing [α-32P] dTTP for 2 hours at 37°C. 30 μl reaction mixes were spotted on pencil labeled DE-81 paper squares (Whatman International, Ltd.). Papers were air-dried again for 30 min and washed 5 times for 10 min with gentle shaking in 100 ml 2 × SSC and twice for 1 min in 50 ml 95% ethanol. Papers were air-dried for 30 min and RT levels were measured by liquid scintillation counting (TRI-CARB 2100TR, PACKARD, USA). All assays were performed in minimum triplicate.

### Real time RT-PCR assay

Total cellular RNA was isolated from cultured U937, primary HFAs using RNeasy Mini Kit (Qiagen) after lysis with TRIzol (Invitrogen) using manufacturer's guidelines. RNA dissolved in DEPC-treated water was used for cDNA synthesis. First-strand cDNA was synthesized by using 500 ng/reaction of the extracted total RNA for subsequent RT-PCR assay as described previously [89]. The prepared first-strand cDNA was diluted 1:1 with sterile water and 5 μl were used per PCR. The primers used in the real-time PCR were as follows: ***GAPDH***: forward primer, 5'-AGCCTTCTCCATGGTGAA; reverse primer, 5'-CGGAGTCAACGGATTTGGTCG; ***TNF-α***: forward primer, 5'-CCCCAGGGCTCCAGAAGGT; reverse primer, 5'-TGGGGCAGAGGGTT GATTAGTTG; ***IFN-α***: forward primer, 5'GGAGGAGAGGGTGGGAGAAAC; reverse primer, 5'-GAAAGCGTGACCTGGTGTATGAG; ***MX1***: forward primer, 5'-CGGGGAAGGAATGGG AATCAGTCA; reverse primer, 5'-TTCCGCACCACGTCCACAACCTT; ***PRKRA: ***forward primer, 5'-GTCCACCAGCCCCATCACAG; reverse primer, 5'-AGGGGCCAGAGGGGAACT TT; ***BST-2***: forward primer, 5'-AGAAGGGCTTTCAGGATGTG; reverse primer, 5'-CTTTTGT CCTTGGGCCTTCT; **HIV-1 *Pol***: forward primer, 5'-TTAAGACAGCAGTACAAATGGCAG T; reverse primer, 5'-ACTGCCCCTTCACCTTTCCA. Semi-quantitative analysis was performed by monitoring in real time the increase of the fluorescence of SYBR Green dye on a Bio-Rad I-Cycler IQ detection system. A threshold cycle value for each gene of interest was determined as previously reported [90]. All data was normalized against the matched *GAPDH *mRNA levels except for U937 electroporation-transfected experiment (Figure 2), which was normalized against the matched *Vpr *mRNA levels.

### Soluble Vpr preparation

The procedure for producing full- length recombinant HIV-1 Vpr protein derived from pNL4-3 has been described previously [91]. In addition, Vpr peptides (70-96) were purchased from Alpha Diagnostic International.

### Calcium imaging

Human neuronal cultures were plated in 35 mm tissue culture dishes (VWR). The 2-5 days old cultures treated with 5 μM Fluo-8 acetoxymethyl ester (Fluo-8-AM) for 30 minutes were imaged as previously described [92]. Changes in Ca^2+ ^induced fluorescence intensity were evoked by glutamate (500 μM, 60 s application) and Vpr peptides and were measured using a confocal microscope equipped with an argon (488 nm) laser and filters (20 × XLUMPlanF1, NA 0.95 objective; Olympus FV300, Markham, Ontario, Canada). Full frame images (512 × 512 pixels) were acquired at a scanning time of 3 s per frame. Selected regions of interest were drawn around distinct cell bodies and traces of time course of change of fluorescence intensity were generated with FluoView v.4.3 (Olympus). Data were only collected from cells in the plane of focus that responded reversibly to a glutamate challenge. Data were collected from 1-5 neurons in each 35 mm dish.

### *In vivo *mouse model

Three-week-old male CD-1 mice were obtained from Charles River Laboratories (Wilmington, MA) and housed in a biocontainment facility according to the guidelines of the Canadian Animal Care Committee. All behavioral testing was performed as described previously [93] by an experimenter blinded to the specific transgenic groups. Animals were placed in a stereotaxic frame under ketamine/xylazine anaesthesia. Full length Vpr (400 μM, 2 μl/each animal), Peptides (20 mM, 2 μl/each animal) and PBS (2 μl) were delivered into the striatum of the animals. *In vivo *neurological injury was assessed according to the Ungerstedt model [94]. In short, ipsiversive rotations as well as total rotations were monitored over 10 min after intraperitoneal injection of amphetamine (1 mg/kg) on days 7, 14, 21, and 28 following striatal injections. More ipsiversive rotations or less total rotations are both indicative of neurological injury. Animals were sacrificed upon completion of the behavioral studies, brains were removed and immersion fixed in 4% paraformaldehyde.

### Tissue preparation and staining

Immunofluorescent labelling was performed using 5 μm paraffin-embedded serial fixed mouse brain sections, prepared as previously described [95]. Briefly, coronal brain sections were deparaffinized and hydrated using decreasing concentrations of ethanol. Antigen retrieval was performed by boiling the slides in 0.01 M trisodium citrate buffer, pH 6.0, for 10 min. Sections were blocked in PBS containing 10% normal goat serum (NGS), 2% bovine serum albumin and 0.1% Triton X-100 overnight at 4°C. The sections were incubated overnight at 4°C with antibodies against ionized calcium binding adaptor molecule (Iba-1; 1:200; Wako, Tokyo, Japan) and glial fibrillary acidic protein (GFAP; 1:200; DAKO, Carpinteria, CA, USA), washed in PBS, then incubated with Alexa 488 conjugated goat anti-rabbit or mouse (1:2500 dilution; molecular probes, Eugene, OR) for 1 hour at room temperature in the dark followed by repeated washing in PBS. The sections were finally mounted with Gelvatol and examined with a Zeiss Axioskop 2 upright microscope (Oberkochen, Germany). The sections for Nissl staining were deparaffinized in 3 changes of xylene and hydrated using decreasing concentrations of ethanol. Slides were then stained in 0.1% cresyl violet solution (Sigma-Aldrich, Oakville, ON, Canada) for 10 minutes and rinsed quickly in distilled water, and differentiated in 95% ethanol. Finally the slides were dehydrated in 100% ethanol, cleared in xylene, mounted with Acrytol (Surgipath, Canada), and examined with a Zeiss Axioskop 2 upright microscope (Oberkochen, Germany).

### Statistical analyses

Statistical analyses were performed using GraphPad InStat version 3.0 (GraphPad Software, San Diego, CA), using ANOVA, for mRNA alterations and neurobehavioral analyses. Unless otherwise stated, all *post-hoc *significant comparisons indicate differences between the control group(s). *p *values < 0.05 were considered significant.

## Abbreviations

HIV: human immunodeficiency virus; Vpr: viral protein R; HAD: HIV-associated dementia; AIDS: Acquired immunodeficiency syndrome; ND: non-demented; PKR: Protein kinase R; HF μϕ: human fetal microglial cells; HFN: human fetal neuron; HFA: human fetal astrocytes; CrFK: Crandle feline kidney; pv: pseudotyped viruses; NGS: normal goat serum.

## Competing interests

The authors declare that they have no competing interests.

## Authors' contributions

CPo and HN conceived and designed the study. HN, assisted by PV and FN, carried out cell transfection, infection, immunostaining and real time RT-PCR experiments; GJ and NB performed patients' sample sequencing; SA carried out calcium imaging studies; FM completed the in vivo experiments; EC made proviral HIV-1 plasmids; HN, SA, FN and FM performed statistical analysis. EC, KB and CPa made substantial contributions to the conception and experimental design of the study, CPo, assisted by HN, SA and FM, wrote the manuscript. All authors have read and approved the final version of the manuscript and have no commercial interests in the manuscript.
